# Second-Order Relational Manipulations Affect Both Humans and Monkeys

**DOI:** 10.1371/journal.pone.0025793

**Published:** 2011-10-03

**Authors:** Christoph D. Dahl, Nikos K. Logothetis, Heinrich H. Bülthoff, Christian Wallraven

**Affiliations:** 1 Physiology of Cognitive Processes Department, Max Planck Institute for Biological Cybernetics, Tübingen, Germany; 2 Human Perception, Cognition and Action Department, Max Planck Institute for Biological Cybernetics, Tübingen, Germany; 3 Language and Intelligence Section, Primate Research Insititute, Kyoto University, Inuyama, Aichi, Japan; 4 Division of Imaging Science and Biomedical Engineering, University of Manchester, Manchester, United Kingdom; 5 Department of Brain and Cognitive Engineering, Korea University, Seoul, Korea; Royal Holloway, University of London, United Kingdom

## Abstract

Recognition and individuation of conspecifics by their face is essential for primate social cognition. This ability is driven by a mechanism that integrates the appearance of facial features with subtle variations in their configuration (i.e., second-order relational properties) into a holistic representation. So far, there is little evidence of whether our evolutionary ancestors show sensitivity to featural spatial relations and hence holistic processing of faces as shown in humans. Here, we directly compared macaques with humans in their sensitivity to configurally altered faces in upright and inverted orientations using a habituation paradigm and eye tracking technologies. In addition, we tested for differences in processing of conspecific faces (human faces for humans, macaque faces for macaques) and non-conspecific faces, addressing aspects of perceptual expertise. In both species, we found sensitivity to second-order relational properties for conspecific (expert) faces, when presented in upright, not in inverted, orientation. This shows that macaques possess the requirements for holistic processing, and thus show similar face processing to that of humans.

## Introduction

In primate societies, a crucial socio-cognitive skill is to recognize and individuate faces. Evolution has provided the primate brain with neural machinery that solves these computationally complex tasks with ease and with great reliability. Two fundamental processes in face processing are (1) the so-called holistic processing and (2) the subordinate-level entry point of faces: Faces share certain features, such as eyes, nose and mouth (featural information), but also a certain configuration of these features (configural information). The term ‘holistic’ refers to the integration of featural and configural information into a single holistic representation [1], [2]. Configural information can be divided further into the so-called first- and second-order relational properties. First-order relational properties describe the general arrangement of features, i.e. the eyes are above the nose, the nose above the mouth, and allow basic-level categorization of faces, i.e. the detection of a face [3]. Second-order relational properties describe the fine-tuned metrics among the features. This information is unique for each individual face and allows classification at the subordinate level, i.e. individuation of faces [1], [2]. By default, the entry-point for faces is at the subordinate level with a face labeled fastest with the name of the individual, e.g. ‘Elvis’, rather than by the basic-level category the face belongs to, i.e. ‘face’. For non-face objects, the opposite case is true: an image of a dog will be labeled as ‘dog’ first, before being labeled by its breed, or its name [4]–[6]. Whether or not these two characteristics, holistic processing and subordinate-level entry point, can be described by the same underlying mechanism remains unclear, however. As pointed out, they share some conceptual aspects of computation. Here, we focus on the aspects related to holistic processing of faces, especially the second order relational properties of facial features [7], i.e. the relative spatial arrangement of facial features. It has been assumed that slight changes in the second order relational properties in a face influence the observer's holistic perception. Slight differences in the spatial arrangement, however, are not explicitly noticeable, but rather result in a “new appearance” of a face. Interestingly, inverting the face [8], [9] seems to disrupt the processing of second order relational properties [7]. In humans, sensitivity to configural manipulations in upright faces has been described in many studies both on the perceptual level [10]–[12] as well as on a memory level [13]. In macaques, however, only a few studies so far have investigated configural sensitivity on a behavioral level [14]–[16]. In Parr et al. [14], a variety of configural (both first- as well as second-order relational manipulations) and featural manipulations were tested in a delayed-matching to sample paradigm using conditions based on an unaltered face, an image of only the inner face, a fractured face, and a fractured and rearranged face. Macaques showed deterioration in performance in all altered conditions, including the inner face condition, allowing no clear-cut interpretation of the results. However, it must be noticed that the ‘fracturing’ manipulation of the stimuli was rather drastic, disrupting the overall appearance of the faces. Our opinion is that sensitivity to configuration must be reflected already at a more subtle stage of manipulation. In Sugita [16], infant macaques without any visual experience in faces were able to detect configural as well as featural manipulations in faces. This finding is surprising and raises the question whether sensitivity to configuration is an innate component, supported by a sensitive period or perceptual narrowing during early infancy, or whether it is a gradual increase due to many years of extensive exposure, as suggested by the human literature [17], [18]. In an adaptation paradigm, Dahl et al. [15] tested macaques on their sensitivity toward slight changes in the inter-ocular distance. A higher rebound of adaptation for the configurally manipulated stimuli as opposed to the normal control stimuli indicating that monkeys are sensitive to configural changes between the eyes.

In summary, while there is some evidence for sensitivity to configural manipulations in macaques, conclusive evidence using carefully manipulated stimuli is still missing. Additionally, and perhaps most importantly, none of the studies so far has directly compared humans and macaques using the *same* task and the *same* stimulus material. In the present study, we set out to address both issues. We first generated face stimuli containing configural manipulations introduced by altering the inter-eye and eye-mouth distances in conspecific faces as well as in non-conspecific faces. In addition, we used both upright and inverted faces. Using these stimuli, we then determined the degree of dishabituation and the proportion of viewing times from eye-tracking of both human and macaque observers in the same task and experimental setting. As in Dahl et al. [15], a habituation-dishabituation paradigm was used together with a preferential looking paradigm that allowed to track changes of interest. Eye gaze was recorded using eye tracking methods. Motivated by the hypothesis that the sensitivity to manipulated spatial relations of the features is disrupted by inversion [19], [20], we hypothesize that with upright faces observers pay more attention to the manipulated facial dimensions (inter-eye and eye-mouth) than with inverted faces. This in turn leads to an increase in viewing times for these parts during the presentation of upright faces as opposed to the inverted faces. We also hypothesize that this enhancement is stronger for conspecific than for non-conspecific faces as a result of the expertise effect [21] and perceptual narrowing [16]. In terms of habituation, we hypothesize that observers show a greater difference in dishabituation for manipulated upright faces versus normal upright faces than for manipulated inverted faces versus inverted normal faces. This effect would reflect a greater dissimilarity between the configurally manipulated version of a face and the normal version when both faces are presented right-side-up - and correspondingly a smaller dissimilarity when presented upside down.

## Methods

### Ethics statement

Participants were recruited from the student population of the University of Tübingen and were paid standard rates of 8€ per hour, or they were affiliates of the MPI. The research presented here consists of a standard monitor psychophysics task with acquisition of eye-tracking data, which falls under standard procedures and hence, no specific ethics approval was sought from the ethics review board. All experiments were conducted in accordance with the 1964 declaration of Helsinki. Before the experiment started, informed, oral consent was obtained from all participants. Furthermore, participants were informed that they could stop the experiment at any time.

This research adhered to the Association for the Study of Animal Behaviour/Animal Behaviour Society Guidelines for the Use of Animals in Research, and the guidelines of the European Community (EU VD 86/609/EEC) for the care and use of laboratory animals under the approval of local authorities (Regierungspräsidium). The animal facilities at the Max Planck Institute for Biological Cybernetics strictly comply with all legal regulations on the use of laboratory animals in research and in many cases sets even higher standards for itself. Only healthy animals living in a stress-free environment can be used in cognitive research. Species-appropriate housing, handling and nutrition are a necessity for conducting behavioral experiments. All animals are kept in mixed groups of young and adult males and females. Climbing furniture and toys as well as places to withdraw are provided as social enrichment. The animal facilities and animal care procedures are regularly monitored by the responsible authorities. On site, a team of experienced veterinarians, biologists and animal caretakers ensure that all animals receive the best possible care. During the experiments, animals are constantly monitored for signs of distress and care is taken to provide a stress-free experimental environment for our behavioral studies. Water and juice rewards are given under ongoing monitoring of our veterinarians, and the daily food rations provide an ample supply of nutrients and fluids.

### Participants

Three rhesus macaques (*Macaca mulatta*, 5 to 7 years old, 10 to 13 kg) and 22 human participants (12 females, age: 18 to 35 years) participated in the study. Prior to the experiments, the monkeys were implanted with a custom-designed titanium head post [22] in a surgical procedure. Macaques were socially housed and had direct and/or visual contact to other colonies and individuals. Contact to humans was restricted, with scientists wearing protective clothes and face masks. Human participants had no explicit knowledge about macaques or related species.

### Stimuli

40 color pictures of rhesus macaque and human faces were used. All faces (macaque and human stimuli) were unfamiliar to both macaque and human participants. Faces were separated from their original background, normalized for luminance, and placed on a mid-gray background in an image canvas of 300×300 pixels (13.3 degrees of visual angle).

**Figure 1:**
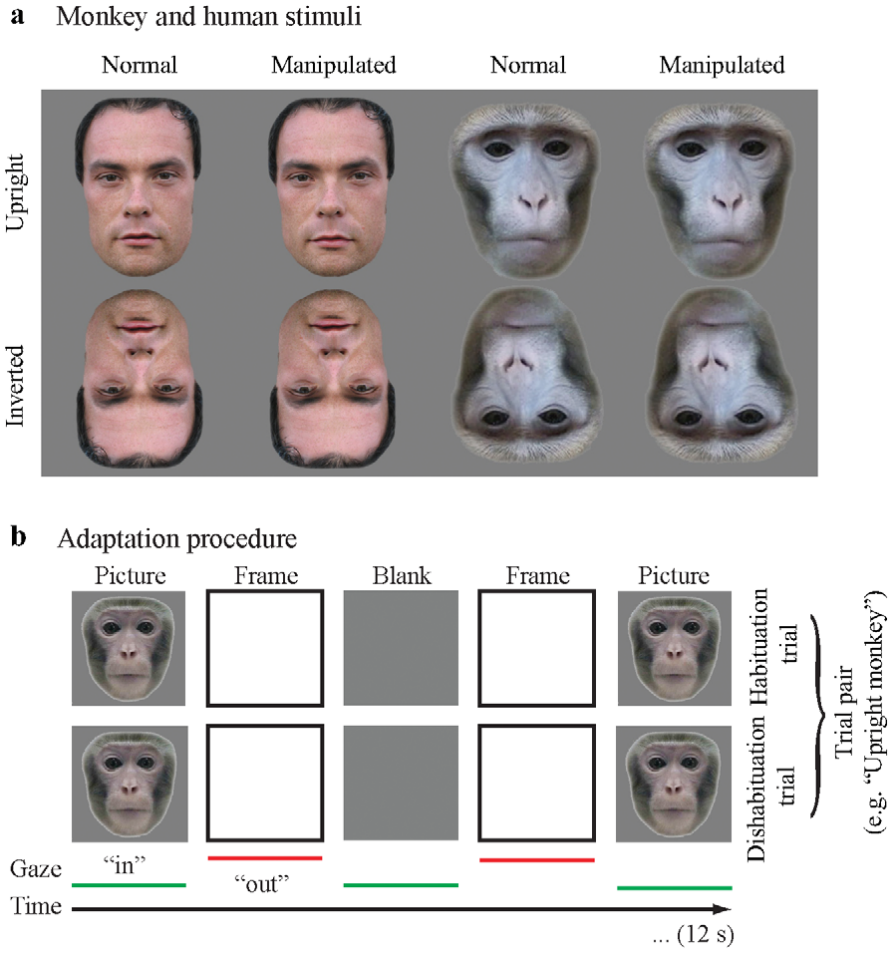
Experiment setup. Panel (a) shows normal and configurally-manipulated stimuli. Face stimuli of humans and macaques were placed on a gray background. Eyes and mouth were spatially displaced. Panel (b) shows the adaptation procedure for an upright macaque trial pair: the first trial (habituation) displays a macaque upright face in alternation with a blank, while the second trial (dishabituation) displays the same macaque upright face configurally manipulated in alternation with a blank. On- and offset of the stimuli are actively controlled by the observer's eye gaze. The ratio between picture and blank reflects the interest in the picture as a function of adaptation/habituation. The rebound of adaptation (dishabituation) reflects the relative rebound of interest in the second picture after having perceived the picture of the first trial. This indicates the perceive dissimilarity in two consecutive pictures.

Stimulus manipulations included whole image rotation for 180 degree (*inverted*), and a configural change of the inter-eye-mouth spacing (*manipulated*). Spacing manipulations of eyes were within a variation of 7–10 pixels, as was the eye to mouth spacing with a variation of 7–9 pixels. The displacement on both dimensions was determined to lie within 2 standard deviations of the mean pixel distances of eight monkeys in our colony as well as eight human faces (see [15]). A mid-gray blank square was used as a gray outline marking a frame of the same size equal to the face stimulus (Figure 1a).

### Procedure and eye tracking

Monkeys were placed in a primate chair inside a darkened sound-attenuating booth during the experiment with head fixation. Stimuli were presented on a 21-inch monitor (Digital, model: VRC21-HA) at a distance of 94 cm controlled by custom-written software under QNX real-time system (QNX Software Systems, Ontario, Canada). Humans were seated in front of a 21-inch monitor (Model: Iiyama Vision Master Pro 21) at a distance of 39 cm inside a darkened experimental room using a chin rest. Both participant groups viewed the stimuli at *equivalent* visual angles (13.3 degrees).

We used an iView infrared eye tracking system (SensoMotoric Instruments (SMI), Teltow/Berlin, Germany) to collect eye movements of the macaques and an iView X™ Hi-Speed infrared eye tracking system to collect human eye movements: both sampled at 200 Hz. A 9-point fixation task was used to calibrate the participant's eye gaze, either prior to each session (macaques) or prior to every trial pair (humans).

The trial order was arranged such that *upright normal* or *inverted normal* trials were followed by *upright manipulated* or *inverted manipulated* trials of the same individual. Macaques did 20+/−3 trial pairs per condition ((upright and inverted)×(human and monkey)) over 10 days of experimental testing. Humans did 20 trial pairs per condition in one experimental session. Statistics were calculated across sessions for monkey participants and across human participants (see [15], [21], [23]).

Participants controlled on- and offset of the stimulus displays by guiding their eye gaze in and out of the central image frame (Figure 1b). Each stimulus display consisted of an alternating picture and blank that were controlled by inwards and outwards eye movements, respectively. The ratio of time the observer spent looking at the picture to the total time spent looking at the picture and the blank (12 seconds) was determined, reflecting the observer's preference for the picture over the blank. With increasing picture exposure, viewing results in habituation. Comparing the habituation of a *normal* trial with a subsequent *manipulated* trial, the *dishabituation* to that second picture after having regarded the first picture can be obtained: This dishabituation is the rebound of interest in the second picture and reflects the perceived degree of similarity between the two consecutively presented pictures. A small rebound indicates similarity; a large rebound indicates dissimilarity between the two faces. The monkeys were rewarded non-specifically with juice for 250–300 ms during an inter-trial interval (5000 ms); humans were financially compensated at standard rates of 8 Euros per hour at the end of the experiment.

### Data analysis

Dependent variables were viewing preference and eye movements. (i.e. the viewing time - we do not report the number of fixations, since the exact same tendency was reflected in that measure). Fixations were defined as a function of velocity, including data samples not faster than 20 deg/s within a time period of at least 100 ms. The final position of that fixation period was determined as the average position of samples during one fixation period. The frequency, density, and duration of fixations on single facial parts (*eyes*, *nose* and *mouth*) were calculated by normalizing the measure for single parts to the total measure in that trial. Also, we subtracted the proportion of the area of each facial part relative to the whole image from the proportion of data samples for each facial part and the total number of samples in that trial. Any deviation from zero therefore means that a facial area was looked at more or less than predicted by a uniform looking strategy. The facial parts ‘eyes’, ‘nose’ and ‘mouth’ were outlined by five humans for all faces using the roi_poly function in Matlab (Mathworks Inc., Natick, MA, USA). The mean of each area across raters was calculated by determining the probability of each pixel being assigned to that area. Pixels exceeding probabilities higher than 0.5 were included in the area templates. Analyses of variances were conducted for the independent variables of stimulus groups (*monkey* versus *human*) as well as for the two stimulus *orientations* (*upright* versus *inverted*) and *stimulus manipulations* (*manipulated* versus *normal*). Corrections for multiple comparisons (alpha/n, where n is the number of comparisons, i.e., a standard Bonferroni correction) were used where applicable. We report the corresponding alpha-level of a single-comparison (e.g. p = 0.05 (reported) is equivalent to p = 0.0167 (tested) for n = 3 comparisons).

## Results

### Preference ratio

**Figure 2:**
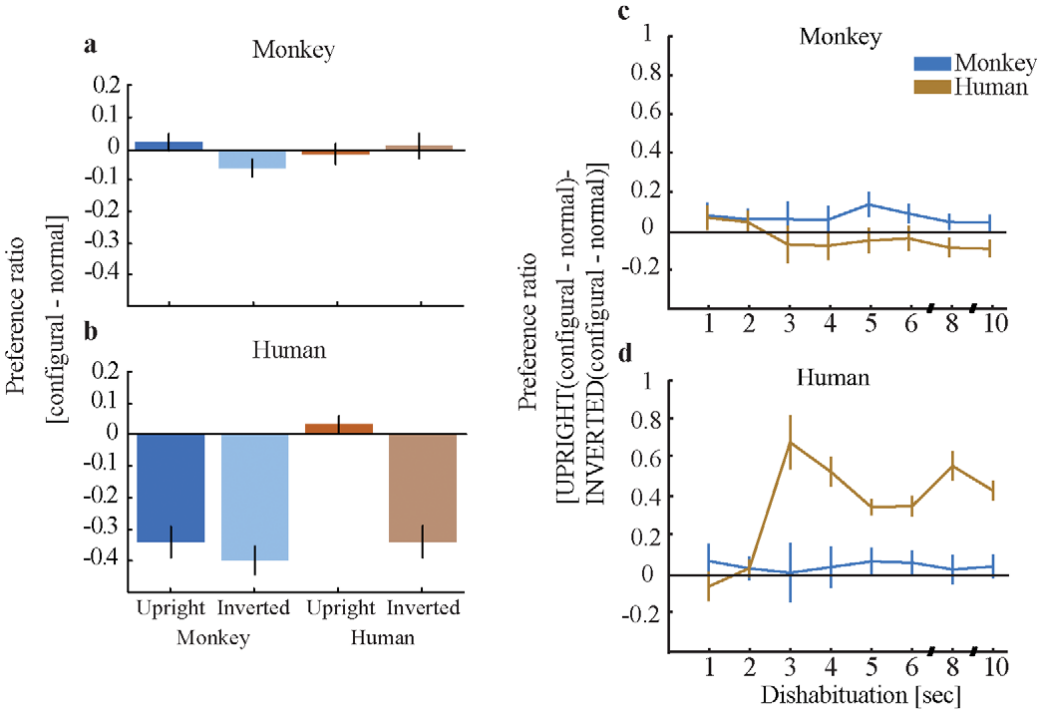
Preference for the face picture above the blank (preference ratio). (a–b) show the grand mean of the difference in preference ratio for configurally manipulated versus normal faces (y-axis) as a function of stimulus species (human versus monkey) and presentation condition (upright versus inverted) (x-axis). Subtitles indicate the species affiliation of the observers. Time course of preference ratio. (c) and (d) show the subtracted values (preference ratio) of the differences between upright configural and normal faces and inverted configural and normal faces as a function of time (sec). The stimulus species is indicated by the line color, the species affiliation of the observers by the subtitles.

The rebound of interest for the configurally manipulated condition relative to the normal condition (i.e. the subtraction of *normal* from *configurally manipulated* conditions) is shown in Figure 2. Values of 0 on the y-axis indicate no additional interest for the *configurally manipulated* condition, while positive values reflect relative interest and negative values relative disinterest. For the monkey observers (Figure 2a), the rebound of interest for the human conditions is at equal level, as indicated by the colored bars: I.e. the rebound of adaptation is orientation insensitive (upright versus inverted) (t(23) = −0.65; p = 0.52; sd = 0.19). The monkey upright condition, however, showed a significant rebound of interest, while the monkey inverted condition resulted in a disinterest in the configurally manipulated stimulus: The rebound of interest for the monkey conditions is significantly different (t(21) = 2.10; p<0.05; sd = 0.19). Conversely, for the human observers (Figure 2b) the rebound of interest for the monkey conditions is similar, as indicated by the color bars: the relative rebound does not depend on the orientation (upright versus inverted) of the face (*t*(19) = 1.72; *p* = 0.10; sd = 0.15). However, the human upright condition showed a large rebound of interest, while the human inverted condition caused a disinterest in the configurally manipulated stimulus (*t*(19) = 5.95; *p*<0.001; sd = 0.28). Time courses for the first 10 seconds of dishabituation are shown in Figure 2c, d.

### Eye tracking analysis

**Figure 3:**
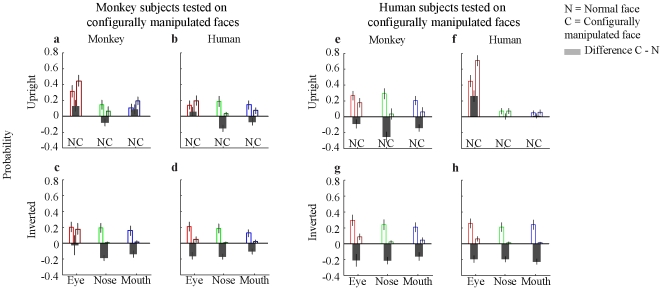
Viewing times of humans and monkeys tested on configurally manipulated faces. Shown are the grand means of looking time, i.e. the time which the observer spent looking at a specific part normalized to the overall looking time and the size of the specific parts. The bars indicate the probability of looking at eyes (red), nose (green) and mouth (blue). ‘N’ stands for the normal face, ‘C’ for the configurally manipulated face. The gray bars show the difference between configurally manipulated and normal faces. a, b, e, f show the upright, c, d, g, h the inverted presentation condition. The subtitles indicate the species of the stimuli. The zero line indicates a random gaze distribution. Values above 0 are of higher probability than random; everything below 0 is of lower probability than random.

**Figure 4:**
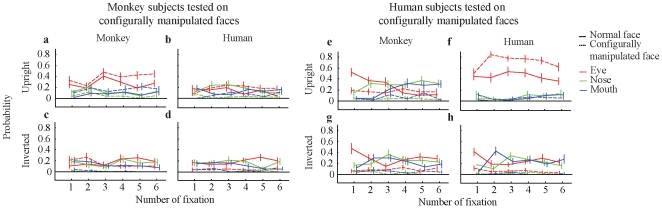
Time courses of monkeys and humans tested on configurally manipulated faces. Panels (a)–(d) show the time courses for monkey observers and panels (e)–(h) for human observers. Facial parts (eyes, nose, mouth) are coded in colors, the manipulation condition (configurally manipulated, normal) in line type (dashed, continuous). The number of fixations are shown on the y-axis, considering the first 6 fixations.

Monkey participants visited the ‘Eyes’ and ‘mouth’ of conspecific upright faces more often in configurally manipulated faces than in normal faces (eyes: *t*(21) = 2.66, *p*<0.01, sd = 0.24; mouth: *t*(21) = 2.15, *p*<0.05, sd = 0.21), while the nose region was visited equally often in configurally manipulated conspecific faces and in normal conspecific faces (nose: *t*(21) = −1.75, *p* = 0.10, sd = 0.21) (Figure 3a). Decreasing interest, however, was observed for the nose and mouth regions of conspecific inverted faces: these facial parts were viewed for a shorter period of time in the configurally manipulated condition than in the normal condition (nose: *t*(21) = −8.86, *p*<0.001 (normal>configural), sd = 0.10; mouth: *t*(21) = −5.04, *p*<0.001 (normal>configural), sd = 0.14) (Figure 3c). The proportion of time spent viewing the eye regions remained constant during the dishabituation trials and is therefore not significantly different (eyes: *t*(21) = −0.44, *p* = 0.66, sd = 0.27) (Figure 3c). For human (non-conspecific) faces the configurally manipulated faces elicited less or no rebound of adaptation and therefore less or equal interest in the manipulated facial parts (Figure 3b, d). This is true for upright faces, reflected in an increase in viewing times for the normal compared to the configurally manipulated condition, (eyes: *t*(23) = 0.77, *p* = 0.45, sd = 0.23; nose: *t*(23) = −4.93, *p*<0.001 (normal>configural), sd = 0.19; mouth: *t*(23) = −1.70, *p* = 0.10, sd = 0.19) (Figure 3b) as well as inverted faces (eyes: *t*(23) = −5.44, *p*<0.001, sd = 0.15; nose: *t*(23) = −7.55, *p*<0.001, sd = 0.12; mouth: *t*(23) = −4.21, *p*<0.001, sd = 0.13, while all normal conditions>configural conditions) (Figure 3d). Figure 4a–d shows the time course of viewing times as a function of number of fixations.

Human participants looked at the eyes of conspecific upright faces more often in configurally manipulated faces than in normal faces (*t*(19) = 4.16, *p*<0.001, sd = 0.26) (Figure 3f). However, the configural changes with respect to the mouth region were apparently too subtle to be reflected in the observers' fixation pattern (*t*(19) = 0.23, *p* = 0.82, sd = 0.09). The unchanged nose region of conspecific faces was visited equally often in the configurally manipulated and in the normal condition and therefore did not reveal a significant difference (*t*(19) = −0.22, p = 0.83, sd = 0.09). The observer's interest decreased when observing conspecific inverted faces (Figure 3h). All facial parts were visited for less time in the configurally manipulated condition than in the normal condition (eyes: *t*(19) = −3.43, *p*<0.01, sd = 0.19; nose: *t*(19) = −6.43, *p*<0.001, sd = 0.14; mouth: *t*(19) = −7.54, *p*<0.001, sd = 0.13). For monkey (non-conspecific) faces the configural manipulation elicited less rebound of adaptation and therefore less interest in the manipulated facial parts (Figure 3e, g). This is true for upright faces (eyes: *t*(19) = −0.37, *p* = 0.72, sd = 0.20; nose: *t*(19) = −6.32, *p*<0.001 (with normal>configural), sd = 0.18; mouth: *t*(19) = −5.08, *p*<0.001 (with normal>configural), sd = 0.12) (Figure 3e) as well as inverted faces (eyes: *t*(19) = −5.19, *p*<0.001, sd = 0.16; nose: *t*(19) = −5.92, *p*<0.001, sd = 0.16; mouth: *t*(19) = −4.01, *p*<0.001, sd = 0.16, with normal>configural) (Figure 3g). The time courses are shown in Figure 4e–h.

### Differences in preference ratios between human and monkey observers

The overall effect size of preference ratio is greater for human than for monkey observers as clearly visible in Figure 2b. However, in both human and monkey observers the critical comparisons between conspecific upright and conspecific inverted faces and the critical similarity of upright non-conspecific and inverted non-conspecific faces are present. The drop of preference ratio in human participants might be due to their belief that the identical picture is presented twice (for the conditions conspecific inverted faces and non-conspecific faces). Comparing the habituation trials (first stimulus) (mean: 0.92; std: 0.08) with the dishabituation trials (second stimulus) (mean: 0.56; std: 0.22) yields a significant effect: *t*(59) = 12.96, *p*<0.001, while this tendency is not apparent for monkey participants (habituation (first) stimulus (mean: 0.43; std: 0.14) versus dishabituation (second) stimulus (mean: 0.42; std: 0.11): *t*(69) = 0.86, *p* = 0.39). Accordingly, quantifying the effect size of preference ratio across participant groups yields a main effect of preference ratio for *Observer* (human versus monkey) (*F*(1,346) = 107.8, *p*<0.001), showing that *overall* the two participant groups look differently. The interactions between the factors *Observer* and *Stimulus* (human versus monkey) (*F*(1,346) = 24.26, *p*<0.001) as well as the interaction between the factors *Observer* and *Stimulus* (conspecific versus non-conspecific) (*F*(1,346) = 36.22, *p*<0.001) were significant. It is important to stress that this *does not* indicate that humans are less interested in these pictures than monkeys; rather, it means that the initial level of interest for humans is much higher for the first picture than for the second picture, which in turn results in a greater relative loss of interest compared to monkeys.

## Discussion

In a recent study by Dahl and colleagues [21], eye movement patterns were modulated by the species affiliation of the presented face. Upright faces of conspecifics contained a high degree of eye salience, i.e. viewing times towards the eyes as opposed to nose and mouth. This eye dominance, however, decreased when faces, irrespective of affiliation, were turned upside down, or when non-conspecific faces were shown. In these conditions, eyes, nose and mouth regions were looked at with equal interest. The eye saliency for the upright face of conspecifics was interpreted as a critical marker for holistic face processing [21]. Recently, this pattern of results obtained with macaques was replicated with chimpanzees (*Pan troglodytes*) [24]. Taken together, the results suggest similar processing mechanisms for face perception across (at least) three primate species. These mechanisms are most efficient when conspecific faces are presented right-side up as neurally encoded schemata of configural and featural information are activated. In addition, we posit that a solid hotspot of fixations, here on the eyes, reflects the involvement of a holistic template including information about the whole face, as opposed to active, serial scanning of facial parts.

Recent work addressed sensitivity of face processing in macaques using the Thatcher illusion [23], [25] in a habituation task. These results illustrate the Thatcher illusion as a function of dishabituation to a thatcherized conspecific face in upright and inverted conditions by eliciting less dishabituation for inverted thatcherized faces (as opposed to the normal inverted face) than for upright thatcherized faces, indicating orientation-sensitive processing of configurations [23], [25]. Moreover, the Thatcher effect was only found for upright conspecific faces, but not for upright non-conspecific or inverted faces [23] providing further evidence for holistic processing expertise that is developed for conspecific faces. A study directly assessing configural sensitivity [14] not only found decreased matching performance for second-order relational manipulations but also for first-order relational manipulations, i.e. the location of features in the face (eyes above nose above mouth, etc.), and for restricted information cues, like the inner features of the face, suggesting that macaques in this study relied on external features. However, their claim that Rhesus monkeys lack expertise in face processing might be problematic, since an alternative strategy of solving a task (as suggested by the authors themselves) does not necessarily exclude the ability of holistic/expert processing under natural conditions. In a study by Dahl and colleagues [15] a greater preference ratio for manipulations on the inter-eye distance was found in three out of four monkeys, suggesting sensitivity to configuration although not entirely robust.

In the present study, we demonstrate a reliable and systematic effect of configural manipulations eliciting a greater rebound of adaptation for conspecific upright faces than for both conspecific inverted or non-conspecific faces. Thus, second-order relational changes are detected well in upright conspecific faces, reflecting the high degree of sensitivity to configural changes in faces. By means of adaptation, a response pattern depending on species affiliation was demonstrated in both humans and monkeys, supporting the view that sensitivity to second-order relational properties is restricted to faces of the viewer's own species and is therefore dependent on the viewer's expertise with the stimulus. In addition we found an enhancement effect, reflected in an increased probability of fixation on the manipulated parts, for configurally manipulated versions of upright conspecific faces.
